# Human Herpesvirus 8 (HHV-8) Staining: A Savior in Early Kaposi Sarcoma

**DOI:** 10.7759/cureus.36486

**Published:** 2023-03-21

**Authors:** Farhan Siddiqui, Mohammed A Al Ameer, Jawad Al-Khalaf, Yusef Al-Marzooq, Ali Al Ameer

**Affiliations:** 1 Department of Laboratory and Blood Bank, King Fahad Hospital, Hofuf, SAU; 2 Department of Dermatology, King Fahad Hospital, Hofuf, SAU

**Keywords:** histopathology, dermatology, immunohistochemistry, hhv-8, kaposi sarcoma

## Abstract

Kaposi sarcoma (KS) is a low-grade vascular neoplasm associated with human herpesvirus 8 (HHV-8) infection. The disease has various phases, and the morphology of the lesion may vary, especially in the early course of the disease, where the morphological features may not be even suggestive of Kaposi sarcoma. The authors take this opportunity to report a case of Kaposi sarcoma where the diagnosis was established because of HHV-8 staining rather than its histopathological features.

## Introduction

In the era of AIDS and solid organ transplantation, some fatal tumors have emerged. Kaposi sarcoma (KS) is one such tumor. KS is a vascular neoplasm, usually low grade but may tend to be locally aggressive. KS has a very strong association with human herpesvirus 8 (HHV-8) infection, to the extent that it is considered a causative agent for the same [1]. KS has three histologic stages: patch, plaque, and tumor/nodular stage [2]. Early KS may have very subtle features and can be easily missed if histological examination does not show the characteristic morphology [3]. The diagnosis of early KS was missed in our case due to the nonspecific findings and the absence of typical histopathological features. Only after HHV-8 staining the diagnosis of KS was established. The authors take this opportunity to augment the importance of HHV-8 staining, especially in early KS, where the histopathological findings are very nonspecific.

## Case presentation

**Figure 1:**
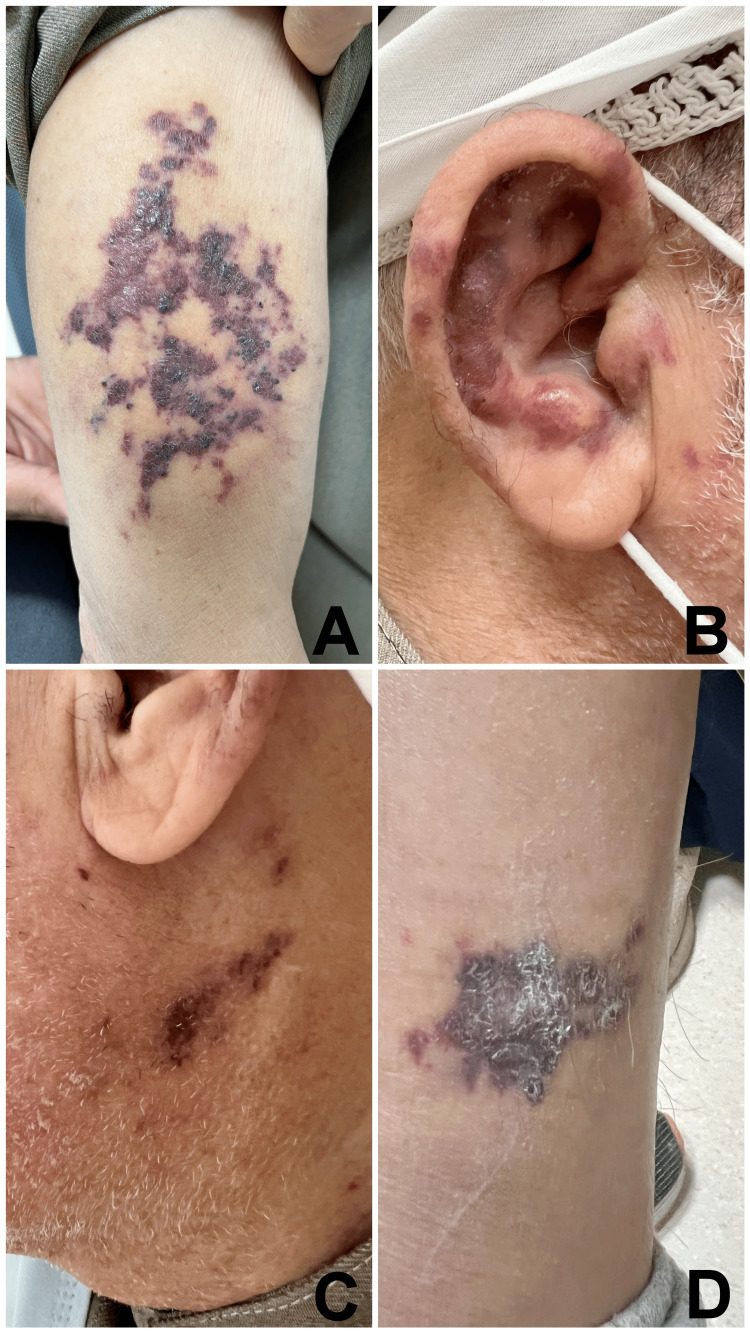
Multiple reddish-bluish patches of variable sizes over the right upper arm (A), right ear (B), left neck (C), and left leg (D)

A 72-year-old male with known type 2 diabetes mellitus (DM), maintaining good control with oral hypoglycemic drugs, was referred to the dermatology department of our hospital. The patient had consulted another dermatology clinic outside our hospital about five months ago. A provisional clinical diagnosis of vasculitis was given there, and a skin punch biopsy was done, which favored the diagnosis of vasculitis. However, there was no response to the treatment, and the patient was referred to our hospital. The patient gave a history of multiple non-pruritic patches over his body at various locations for one year. On examination, the patient was well oriented with normal vitals (pulse of 72/minute, respiratory rate of 14/minute, and blood pressure of 124/82 mm Hg). Local examination showed multiple reddish-bluish patches of variable sizes over the right upper arm, both ears, and lower legs, with some having nodular configurations (Figure 1).

The process started as small red dots (puncta) macules and papules, which later clustered together to form a linear or serpiginous pattern. Oral mucosa was uninvolved. No organomegaly or lymphadenopathy was identified. There was no history of any discomfort with swallowing, swollen legs, respiratory or gastrointestinal symptoms. A clinical differential diagnosis of Kaposi sarcoma was suggested.

**Figure 2:**
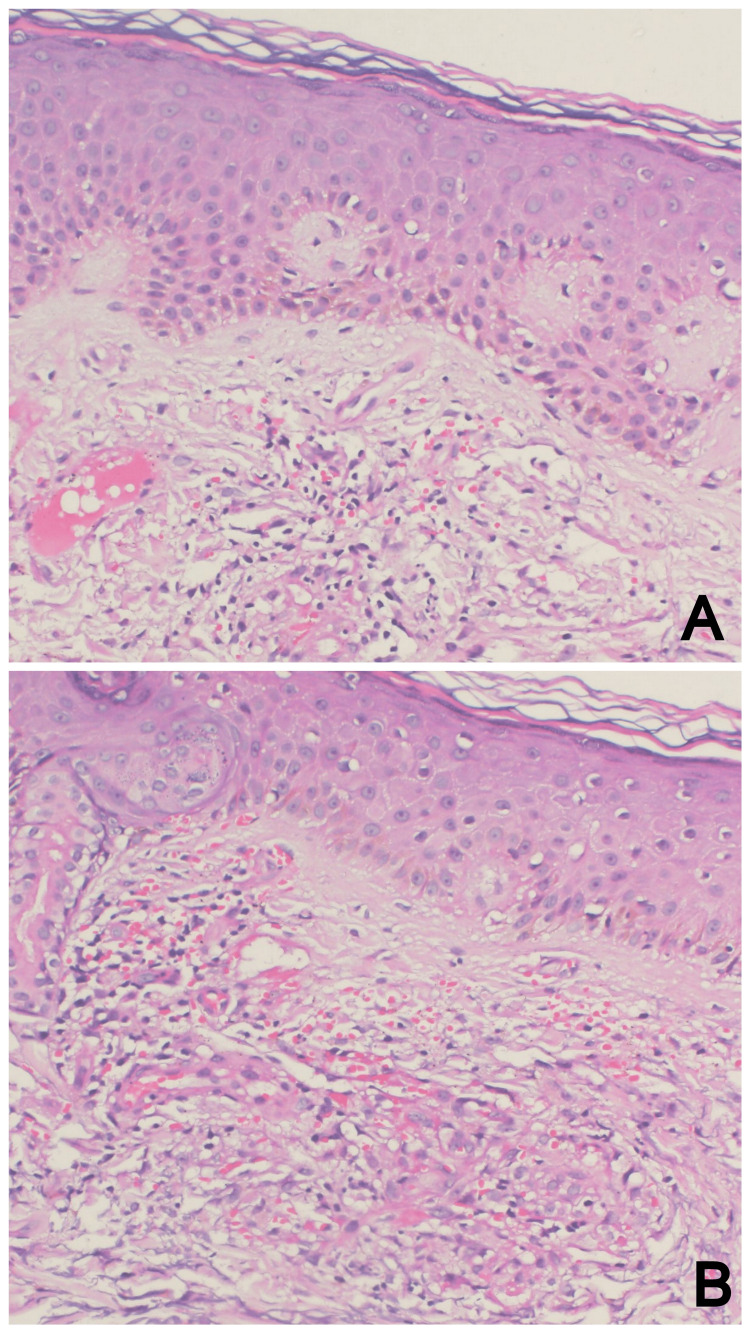
Microphotographs (A and B) showing superficial dermal perivascular moderate inflammatory infiltrate with extravasation of red blood cells (H&E, 200x)

The previous biopsy slides were retrieved from the dermatology clinic outside our hospital and were then reviewed intra-departmentally at our hospital, and none of the histopathologists favored Kaposi sarcoma. The H&E stained slides showed superficial dermal perivascular moderate inflammatory infiltrate with extravasation of red blood cells (RBCs; see Figure 2).

**Figure 3:**
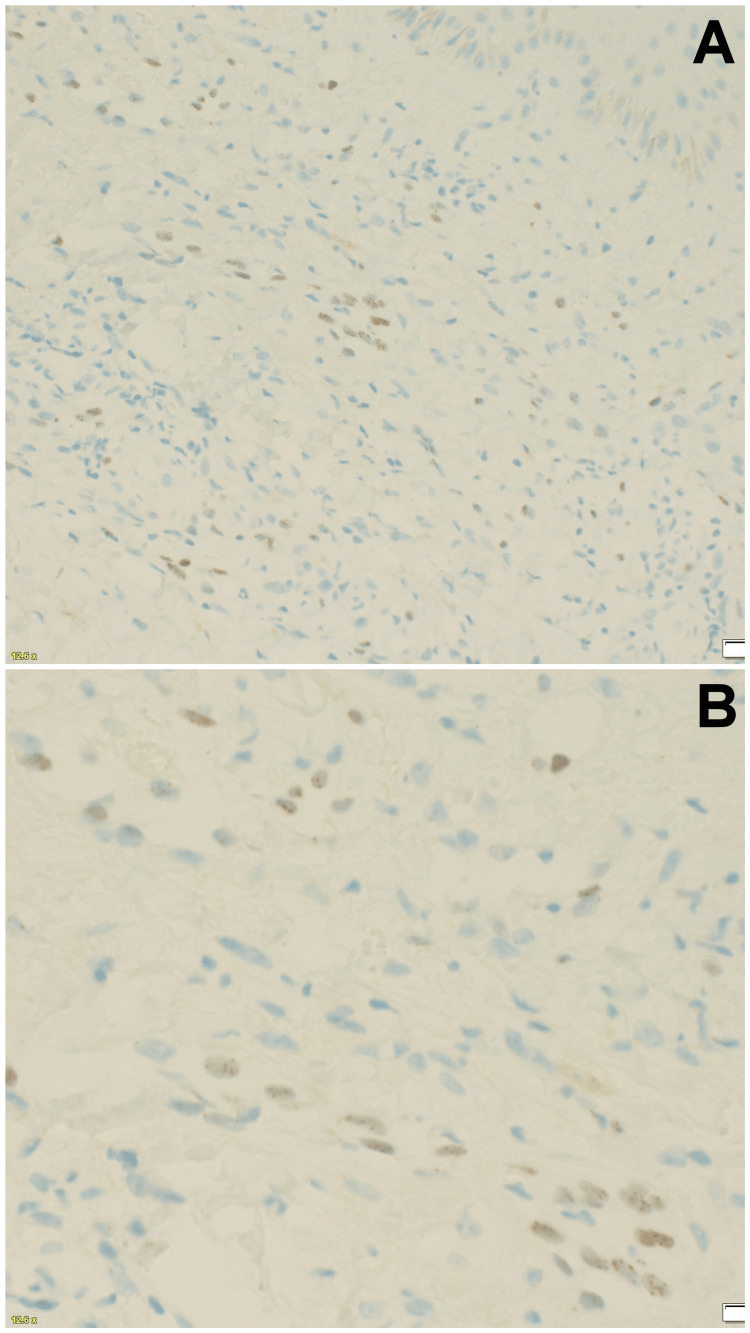
Microphotograph (A) showing scattered positivity in some cells (HHV-8, 200x) and microphotograph (B) showing nuclear dot-like positivity (HHV-8, 400x) HHV-8 - human herpesvirus 8

In view of strong clinical suspicion for Kaposi sarcoma, HHV-8 staining was requested, with the plan for a repeat biopsy as a backup. HHV-8 staining was found to be positive (Figure 3). The possibility of angiosarcoma was also questioned; however, positive HHV-8 staining ruled out angiosarcoma. An amended report was given, with features consistent with Kaposi sarcoma. The patient was also tested for HIV and was found to be negative. Topical therapy in the form of imiquimod 5% cream was applied under occlusion three times a week for 12 weeks duration without significant improvement. The patient was referred to a specialist hospital and oncology center for further evaluation regarding the choice of management.

## Discussion

In 1872, Moritz Kaposi, a dermatopathologist from Hungary, had reported an aggressive "idiopathic multiple pigmented sarcoma" arising in the skin. This sarcoma was later designated as Kaposi sarcoma [3]. Kaposi sarcoma (KS) is a vascular neoplasm, often arising in multiple parts of the body, and is characterized by four clinical/epidemiologic manifestations: classic, iatrogenic, endemic (African), and associated with AIDS [4,5]. Mortiz Kaposi has described the classic type of KS, which is usually seen in elderly people (>50 years) with male predilection in the Mediterranean region, Eastern Europe, and the Middle East [6]. The iatrogenic variant has been linked to organ transplantation, auto-immune diseases, and the use of corticosteroids or long-term cytotoxic drugs. It is generally seen within two years of therapy [6]. In endemic (African) KS, patients are usually males and younger (<40 years). In AIDS-associated KS, homosexual and bisexual individuals are high-risk groups [7].

KS usually involves the skin and lymph nodes, but other sites have also been documented, including mucosa (oropharynx and digestive tract) and visceral organs [8]. Clinically, the lesions initially commence as blue-red macules that may evolve into plaques and then eventually develop into nodules. The clinical picture and histopathologic features of the four epidemiologic forms are very much alike, with all progressing in the same pattern from early patch to plaque and eventually nodular stage. At each stage, KS has significant morphologic overlap with other vasoproliferative lesions. The microscopic features of KS are synonymous with the stage of KS rather than the clinical setting [9]. Histopathologically, cutaneous KS shows dermal proliferation of small vessels usually surrounding larger ectatic vessels, with slit-like vascular spaces lined by endothelium and spindle-shaped cells accompanied by sparse lymphoplasmacytic inflammatory infiltrate along with hemosiderin pigment and haematic extravasation. Hyaline globules (extracellular and intracytoplasmic) may also be seen. The population of the dermal spindle cells increases progressively with the stage of the lesion. The early lesions (patch and plaque stage) may show very subtle nonspecific features on histology, sometimes may even mimic granulation tissue, and therefore can be easily missed. The advanced nodular lesions may be composed, of spindle cells predominantly, raising the possibility of cellular spindle cell neoplasms, likely fibrosarcoma [3]. Features that favor KS are the presence of interlacing fascicles of spindle cells, diffuse distribution of haematic extravasation with hemosiderin, exophytic and endophytic growth pattern, lack of septae, and absence of a lobulated growth pattern [9].

HHV-8 DNA has been detected in virtually all forms of KS but not in the perilesional unaffected skin of patients with KS [3]. HHV-8 is negative in other vascular tumors with which KS shares histologic features [9]. The presence of HHV-8 is now believed to be a major commencing event for the development of KS. It is detected in the spindle cells and endothelial cells of KS, some but not all authors are of the opinion that HHV-8 has been found in tumor leukocytes as well [8]. About 5% of the world's population is infected with HHV-8, but it does not correlate with the incidence of KS (<1/100,000) [10]. The mere positivity of HHV-8 does not concur with the development of KS, but progression relies on some degree of the host immune system and the local inflammatory milieu [11].

Using polymerase chain reaction (PCR), HHV-8 is found in about 95-100% of Kaposi's sarcoma lesions [10]. However, it has also been described in two AIDS-related lymphoproliferative disorders - primary effusion lymphomas and multicentric Castleman's disease (MCD). About 75% of patients with MCD develop KS [7,12]. However, some data also shows HHV-8 positivity in myofibroblastic inflammatory tumors, a few non-Hodgkin lymphomas, and Hodgkin lymphoma [8].

The histological diagnosis may also be further verified by immunohistochemistry (IHC) for HHV-8 latent nuclear antigen-1 (LNA-1). LNA-1 helps viral DNA bind to the host chromosomes, and hence immunoreactivity stains in the pattern of nuclear stippling. The IHC for HHV-8 is considered positive when the staining is nuclear with a granular appearance and has no concomitant cytoplasmic staining [8]. HHV8 IHC plays a major role in establishing the diagnosis of KS, especially when clinical features favor KS but the histological morphology mimics other vasoformative lesions (such as pyogenic granuloma and angiosarcoma) in subtle patch stage KS or does not favor KS [13], as was seen in our case.

The detection of HHV-8 in tumor cells can be done using reverse transcriptase (RT)-PCR as well as by immunohistochemistry. IHC is clearly less labor-intensive and time-consuming, easier, inexpensive, less complex, and more practical as it allows one to correlate with the morphologic features and definitely less prone to false positive results or erroneous interpretations in view of contamination, in comparison to RT-PCR [8,9]. Therefore, RT-PCR is not favored to be performed alone but always in collaboration with competent microscopic and immunohistochemical examination. However, PCR for HHV-8 should be considered when encountering clinical or histological features suggestive of KS with negative HHV-8 immunohistochemistry [14].

Therapeutic options for KS are variable, including local therapy like topical imiquimod or cryotherapy, radiation therapy, chemotherapy, and immunotherapy. The appropriate selection for each patient depends on the general health of the patient as well as the number, location, and size of the KS lesions [15]. 

## Conclusions

Early Kaposi sarcoma may not show the typical characteristic histological picture but rather reveal nonspecific features. This may create a dilemma for the pathologist as well as the dermatologist and eventually lead to a delay in the diagnosis of KS and further proper management. HHV-8 immunostaining is 99% sensitive and 100% specific for the diagnosis of KS, and therefore cases where histological features do not favor KS, as was seen in our case, HHV-8 immunostaining can play a vital role in early diagnosis and treatment. However, it is not practical to do HHV-8 staining in all skin biopsies without any context. Clinical suspicion of Kaposi sarcoma is definitely required to go forward with HHV-8 staining in cases with nonspecific histopathological findings.
